# Chemical Constituents and Pharmacological Activity of Agarwood and *Aquilaria* Plants

**DOI:** 10.3390/molecules23020342

**Published:** 2018-02-07

**Authors:** Shuai Wang, Zhangxin Yu, Canhong Wang, Chongming Wu, Peng Guo, Jianhe Wei

**Affiliations:** 1 Key Laboratory of Bioactive Substances and Resources Utilization of Chinese Herbal Medicine, Institute of Medicinal Plant Development, Chinese Academy of Medical Sciences & Peking Union Medical College, Beijing 100193, China; zhuizhirun@163.com; 2 Ministry of Education & National Engineering Laboratory for Breeding of Endangered Medicinal Materials, Institute of Medicinal Plant Development, Chinese Academy of Medical Sciences & Peking Union Medical College, Beijing 100193, China; 3 Conservation and Development of Southern Medicine, Hainan Branch of the Institute of Medicinal Plant Development, Chinese Academy of Medical Sciences & Peking Union Medical College, Haikou 570311, China; yu_xin712@163.com (Z.Y.); xinzhuangjianpo@163.com (C.W.); 4 Key Laboratory of State Administration of Traditional Chinese Medicine for Agarwood Sustainable Utilization, Hainan Branch of the Institute of Medicinal Plant Development, Chinese Academy of Medical Sciences & Peking Union Medical College, Haikou 570311, China; 5 Pharmacology and Toxicology Center, Institute of Medicinal Plant Development, Chinese Academy of Medical Sciences & Peking Union Medical College, Beijing 100193, China; cmwu@implad.ac.cn

**Keywords:** agarwood, *Aquilaria* plants, chemical constitutes, bioactive compounds, pharmacological function

## Abstract

Agarwood, a highly precious non-timber fragrant wood of *Aquilaria* spp. (Thymelaeaceae), has been widely used in traditional medicine, religious rites, and cultural activities. Due to the inflated demanding and depleted natural resources, the yields of agarwood collected from the wild are shrinking, and the price is constantly rising, which restricts agarwood scientific research and wide application. With the sustainable planting and management of agarwood applied, and especially the artificial-inducing methods being used in China and Southeast Asian countries, agarwood yields are increasing, and the price is becoming more reasonable. Under this condition, illuminating the scientific nature of traditional agarwood application and developing new products and drugs from agarwood have become vitally important. Recently, the phytochemical investigations have achieved fruitful results, and more than 300 compounds have been isolated, including numerous new compounds that might be the characteristic constituents with physiological action. However, no one has focused on the new compounds and presented a summary until now. Alongside phytochemical advances, bioactivity screening and pharmacological investigation have also made a certain progress. Therefore, this review discussed the new compounds isolated after 2010, and summarized the pharmacological progress on agarwood and *Aquilaria* plants.

## 1. Introduction

Agarwood, known as chenxiang in Chinese and called aloeswood, agalloch, eaglewood, jinkoh, gaharu, or kanankoh in different regions, is a highly valuable non-timber fragrant wood of *Aquilaria* spp. (Thymelaeaceae) [1,2,3,4]. There are 31 species of *Aquilaria* found worldwide in Indonesia, Malaysia, China, India, Philippines, Cambodia, Vietnam, Laos, Thailand, Papua New Guinea, and Singapore [5,6], among which 19 species can produce agarwood after being attacked by physical force [7,8], insects [9], or bacteria/fungi infection [10,11,12]. Agarwood is used for incense, perfume, traditional medicine, and other products in the world market. In traditional Chinese medicine, agarwood is used as a *qi*-regulating drug and carminative medicine to relieve gastric problems, coughs, rheumatism, and high fever. It can promote *qi* circulation to relieve pain, warm the middle energizer to arrest vomiting, and regulate respiration to relieve asthma [13]. In traditional Arabian medicine, agarwood essential oil is used for aromatherapy. Simultaneously, agarwood has also been widely used for centuries as incense in Buddhist, Hindu, and Islamic ceremonies.

With the increasing demand for agarwood, the population of *Aquilaria* species is declining rapidly in the wild, and all species of *Aquilaria* have been placed on the Appendix II list of the Convention on International Trade in Endangered Species of Wild Fauna and Flora since 2004 [1]. In response to this situation, sustainable planting and management of agarwood with artificial methods are arising, and the agarwood yield is increasing. As a result, agarwood no longer needs to be obtained from wild natural resources, enabling its wider application and investigation, especially on pharmaceutical study. Based on the phytochemical studies, a number of new compounds have been isolated and identified from agarwood and *Aquilaria* plants. However, there is no literature concentrating on the new compounds, even though earlier literature have summarized the chemical constituents of agarwood and related plants [6,14,15]. Therefore, this review discussed the new compounds isolated after 2010, and summarized the pharmacological progress on agarwood and *Aquilaria* plants.

## 2. Results and Discussion

### 2.1. New Compounds from Agarwood and Aquilaria Plants

The chemical constitutes of agarwood originating from the genus *Aquilaria*, include 2-(2-phenylethyl)-4*H*-chromen-4-one derivatives, terpenoids, flavonoids etc., in which 2-(2-phenylethyl)-4*H*-chromen-4-one derivatives and sesquiterpenes are the two predominant constituents in agarwood. There have been 154 new compounds (Table 1) isolated from agarwood and genus *Aquilaria* trees since 132 compounds were summarized in June 2010 [14].

#### 2.1.1. 2-(2-Phenylethyl)chromones

**Figure 1:**
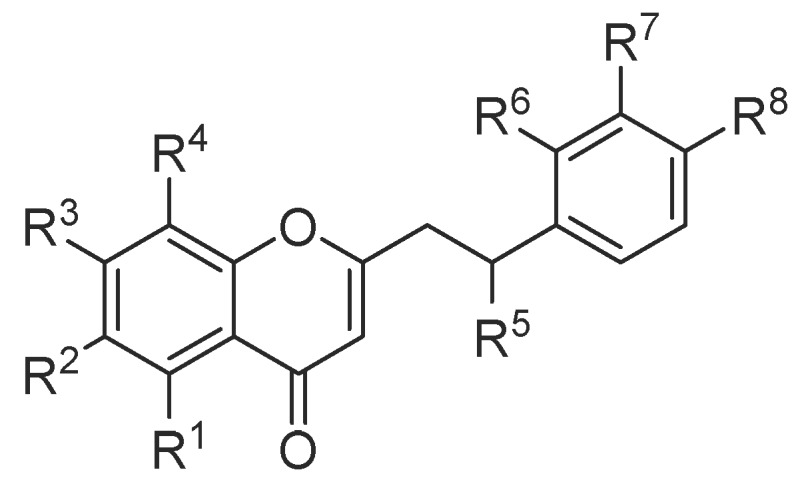
Structures of chromones identified in agarwood.

**Figure 2:**
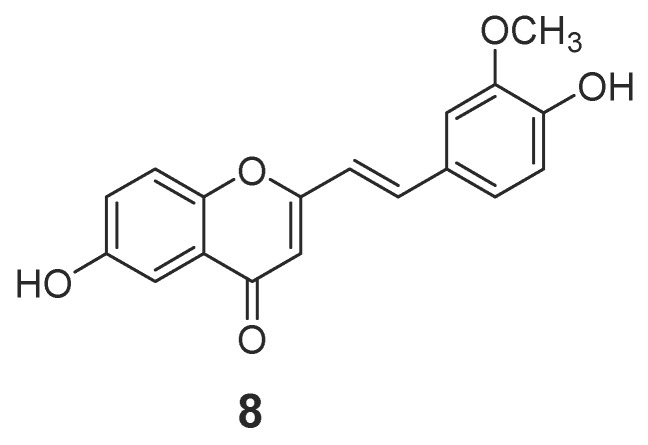
Structure of 2-(2-phenylethenyl)chromone identified in agarwood.

**Figure 3:**
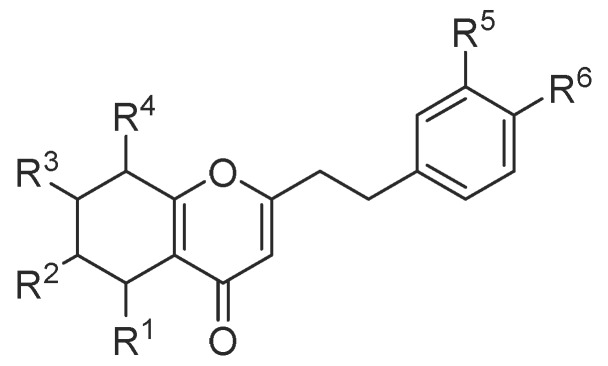
Structures of 5,6,7,8-tetrahydro-2-(2-phenylethyl)chromones identified in agarwood.

**Figure 4:**
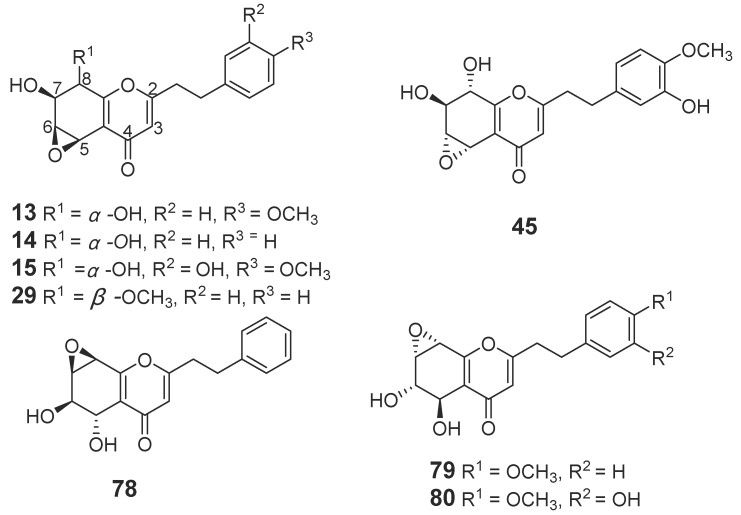
Structures of 5,6,7,8-tetrahydro-2-(2-phenylethyl)chromones with epoxide identified in agarwood.

**Figure 5:**
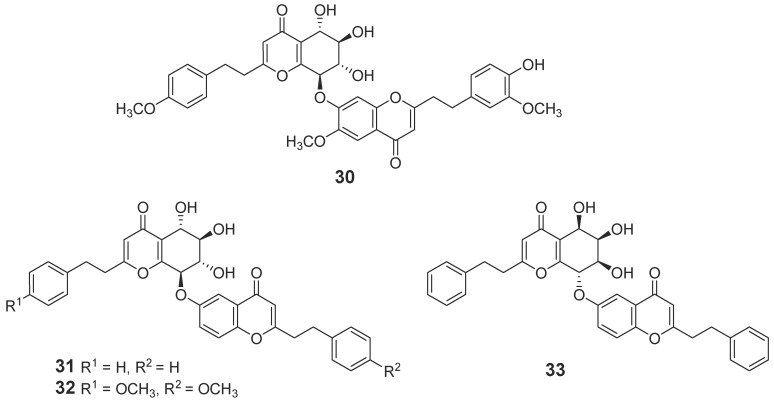

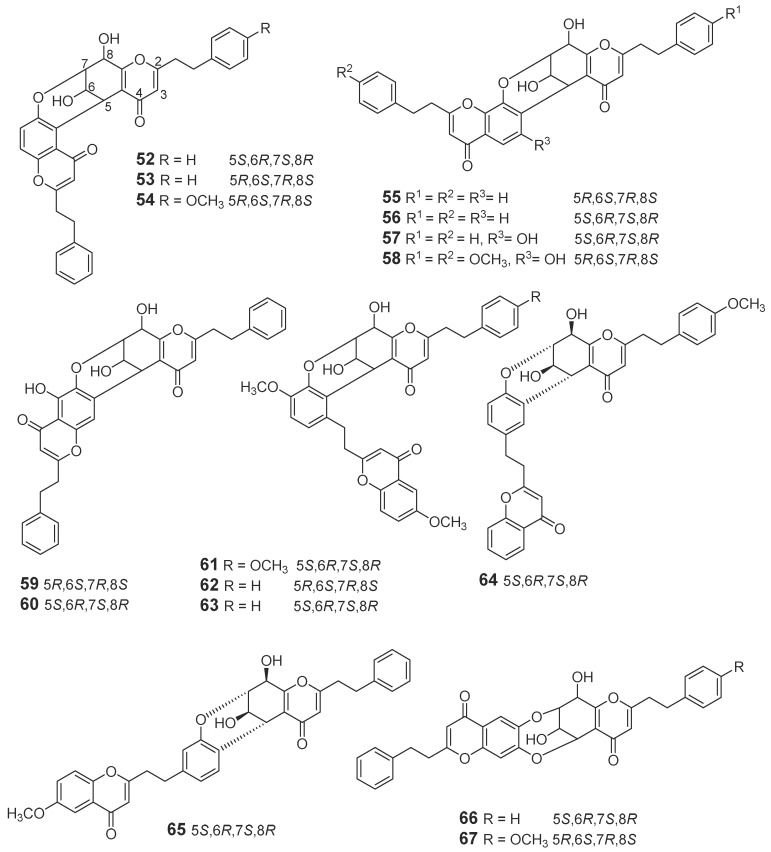
Structures of dimeric 2-(2-phenylethyl)chromones identified in agarwood.

In total, 88 new 2-(2-phenylethyl)chromone compounds (**1**–**88**) have been isolated from agarwood and genus *Aquilaria* plants (Figure 1, Figure 2, Figure 3, Figure 4 and Figure 5). Yang et al. [16] carried out a bioassay-guided isolation strategy from *A. sinensis*, resulting in seven new 2-(2-phenylethl)chromone derivatives **(1**–**7**) and a new 2-(2-phenylethenyl)chromone (**8**) being obtained from an ethanol (EtOH) extract. The investigation of EtOH extract obtained another three 2-(2-phenylethl)chromones (**9**–**11**) [17,18] and eight derivatives (**12**–**19**) from different fractions [19]. Liao et al. [20] reported seven new 2-(2-phenylethyl)chromone derivatives (**20**–**26**), including a chlorinated one (**23**) from the ethyl acetate (EtOAc) fraction of artificial agarwood (*A. sinensis*). The EtOAc fraction also contained three 2-(2-phenylethyl)chromones (**27**–**29**) [8] and four new bi-phenylethylchromones (**30**–**33**) [21]. A phytochemical investigation of a resinous wood (*A. sinensis*) led to the isolation of nine new 2-(2-phenylethyl)chromone derivatives, aquilarones A–I (**34**–**42**) from a chloroform (CHCl_3_) fraction [22]. Liu et al. found four new 2-(2-phenylethyl)chromone derivatives (**43**–**46**) from Chinese agarwood produced via the whole-tree agarwood-inducing technique [23]. Huo et al. gained five new 2-(2-phenylethyl)chromone derivatives (**47**–**51**) [24] and sixteen dimeric 2-(2-phenylethyl)chromones (**52**–**67**) from the resinous wood of *A. sinensis* [25]. Liao et al. isolated thirteen 5,6,7,8-tetrahydro-2-(2-phenylethyl)chromones (**68**–**80**) [26] from the artificial agarwood of *A. sinensis.* Additionally, one 2-(2-phenylethyl)chromone compound (**81**) was isolated from the stem bark EtOH extract of *A. sinensis* [27].

“Qi-Nan” is regarded as the highest quality agarwood, valued for its mysterious oriental odor that can be smelt without burning, unlike other kinds of agarwood. The investigation of EtOH extract of high-quality Chinese agarwood “Qi-Nan” (*A. sinensis*) obtained seven new 2-(2-phenylethl)chromone derivatives (**82**–**88**) [28,29].

#### 2.1.2. Terpenoids

**Figure 6:**
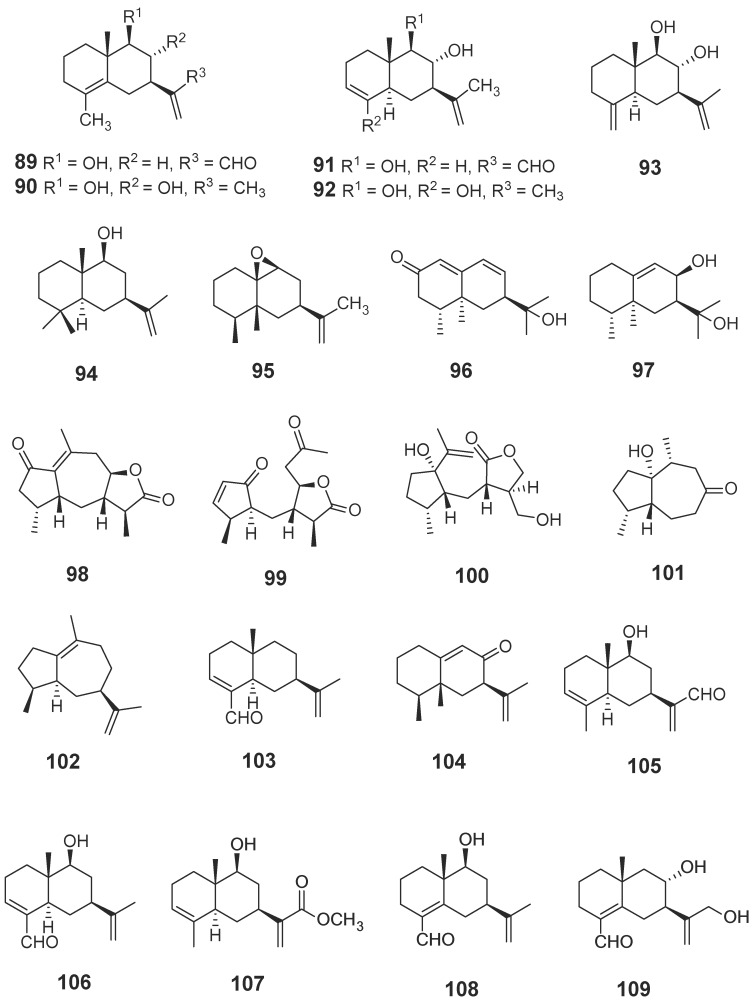

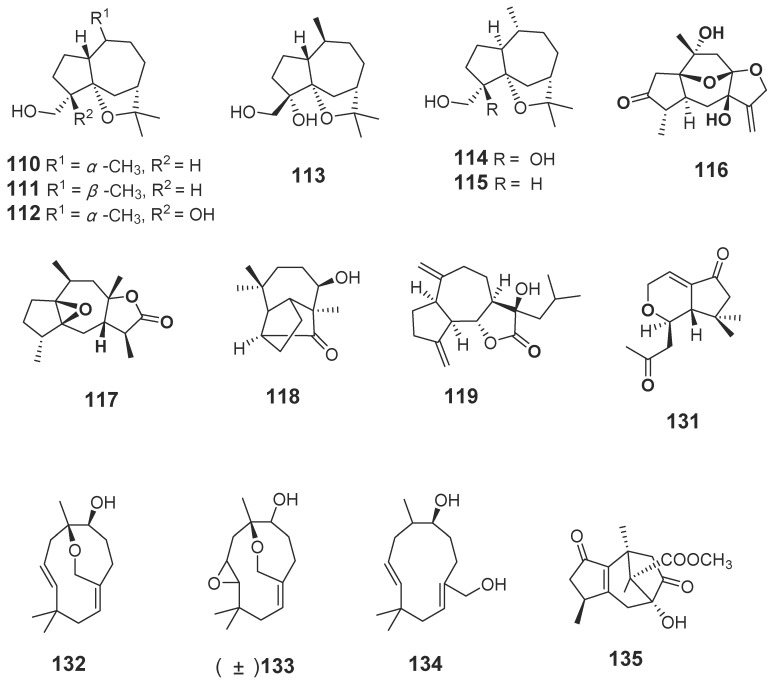
Structures of sesquiterpenes identified in agarwood.

**Figure 7:**
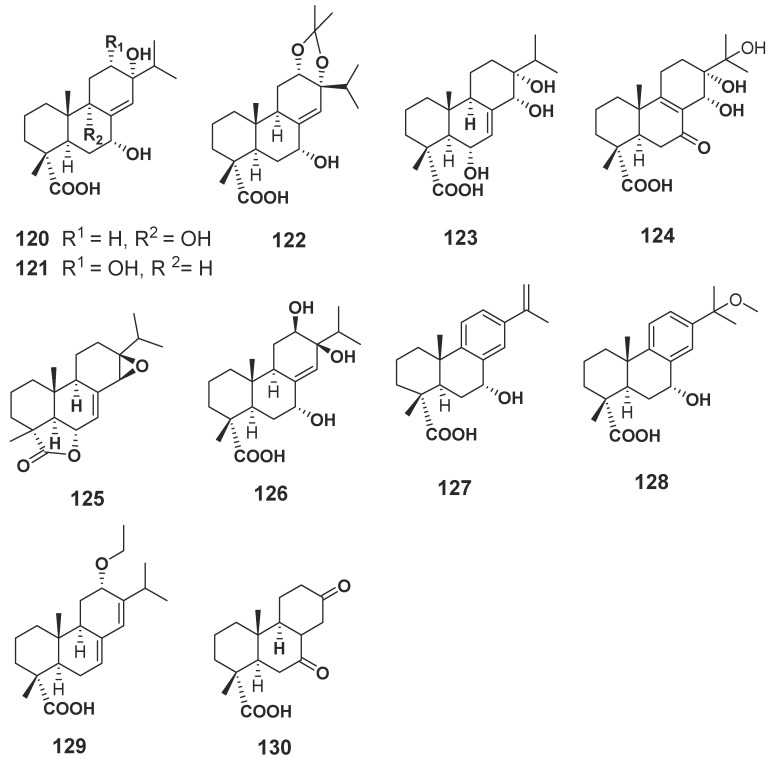
Structures of diterpenes identified in agarwood.

**Figure 8:**
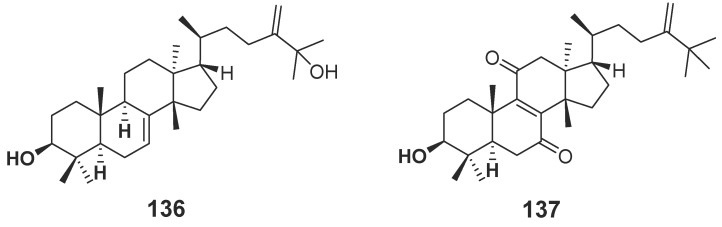
Structures of tirucallane triterpenoids from *Aquilaria sinensis.*

**Figure 9:**
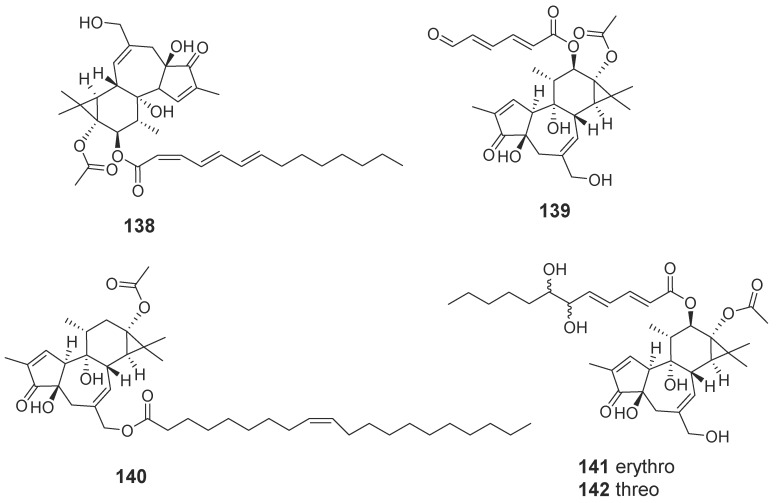
Structures of phorbol esters from *Aquilaria malaccensis.*

Terpenoids are compounds derived from mevalonic acid, whose basic carbon frame is characterized by having two or more isoprene units. Terpenoids, including sesquiterpenes and diterpenes, are the main components of agarwood. The EtOH extract of agarwood was isolated and, as a result, a total of 34 new sesquiterpenes (**89**–**117**, **131**–**135**) (Figure 6) were gained [30,31,32,33,34,35,36], in which nine compounds (**102**–**104**, **110**–**115**) were identified from “Qi-Nan” [32,34]. The isolation of a petroleum ether fraction obtained two new sesquiterpene derivatives (**118**, **119**) (Figure 6) [37], and eleven new diterpenoids (**120**–**130**) (Figure 7) were identified from EtOH extract [38]. Additionally, many new terpenoids have also been found in other parts of genus *Aquilaria* plants. Peng et al. [39] isolated a novel degraded sesquiterpene, named aquilarin B (**131**) (Figure 6) from the EtOH extract of the fresh stem (*A. sinensis*) and Cheng et al. [40] got two new tirucallane triterpenoids (**136**–**137**) (Figure 8) from the leaves of *A. sinensis*. Furthermore, aquimavitalin (**138**) and four new phorbol esters (**139**–**142**) were isolated from an *A. malaccensis* seeds ethanolic extract [41,42] (Figure 9).

#### 2.1.3. Flavonoids

**Figure 10:**
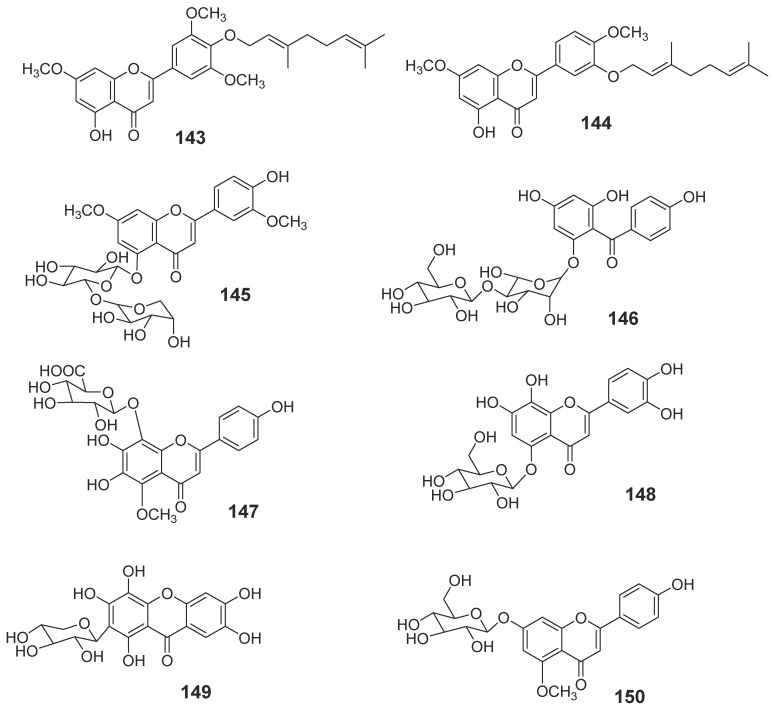
Structures of flavonoids from *Aquilaria malaccensis.*

Flavonoids consist of a large group of polyphenolic compounds with a benzo-γ-pyrone structure, which is ubiquitously present in plants; there is no exception for the genus *Aquilaria* plants. Two new flavones (**143**, **144**) were obtained from the EtOAc fraction of stem bark (*A. sinensis*) [27] (Figure 10). Another six new flavonoids (**145**–**150**) were isolated from the leaves of *A. sinensis* [43,44,45] (Figure 10).

#### 2.1.4. Others

**Figure 11:**
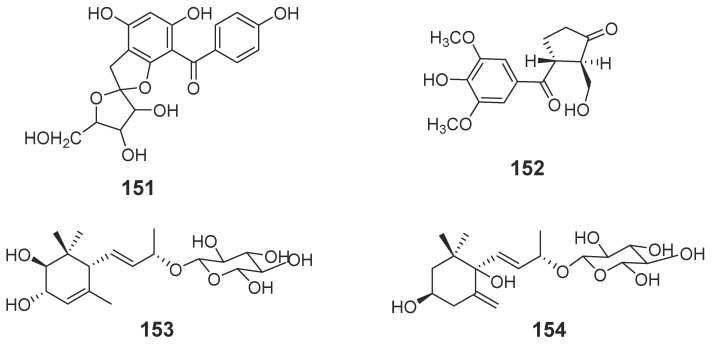
Structures of compounds from agarwood.

Compounds **151**–**154** are included here, as they do not belong to any of the above classes [45,46,47] (Figure 11).

### 2.2. Pharmacological Activity of Fraction and Components from Agarwood and Aquilaria Trees

#### 2.2.1. Neural Activity

Agarwood has been traditionally used as a medicine for tranquilizing and reducing excitement in China, Southeast Asia, and the Middle East for centuries. Modern pharmacological studies have demonstrated that agarwood has an active effect on the nervous system [48,49]. Okugawa et al. [50] determined that a benzene extract of *A. malaccensis* agarwood reduced spontaneous motility, prolonged hexobarbiturate-induced sleeping time, and decreased rectal temperature, whereas petroleum ether, chloroform, or water extracts did not have that effect. A further bio-guided isolation of a benzene extract found that jinkoh-eremol and agarospirol were the main active constituents [51,52]. Takemoto et al. [53] reported that agarwood essential oil sedated mice through vapor inhalation, in which the main volatile constituents were benzylacetone, *α*-gurjunene, and (+)-calarene. As benzylacetone had a sedative effect, a number of derivatives were synthesized and assessed for a sedative effect. The results demonstrated that benzylacetone-like compounds had sedative activities, and their intensities varied depending on the functional group in the carbon chain, the substituent in the benzene ring, and their combinations [54]. Our recent studies showed that both the ethanol extract and essential oil of agarwood, induced by the whole-tree agarwood inducing technique in *A. sinensis* trees, had a sedative-hypnotic effect, where its potential mechanism is related to regulating the gene expression of GABA_A_ receptors and potentiating the GABA_A_ receptor function [55,56]. Agarofuran, derived from agarwood essential oil, was reported to have anxiolytic and anti-depression activity in mice [49]. To explore a potential drug for treating anxiety and depression, a series of agarofuran-like derivatives were synthesized and the activity screened, among which, buagafuran was an effective compound for anti-anxiety and anti-depression, with low toxicity and a high safety coefficient [49,57]. The potential mechanism might be through modulating central neurotransmitters, such as dopamine [58]. A metabolic study showed that buagafuran could be transformed to hydroxy metabolite and carbonyl one in a human liver microsome, where carbonyl metabolite was the main one [59]. Until now, phase II clinical trials are being conducted on buagafuran. Furthermore, many other activity screening results have also shown that compounds from agarwood have an effect on neural activity. Compound **7** (10 µg/mL) showed neural protective activity against both glutamate-induced and corticosterone-induced neurotoxicity in PC12 pheochromocytoma and human U251 glioma cells [16]. Compounds **118** and **119** exhibited potent anti-depressant activity in vitro by inhibiting [^3^H]-5-HT reuptake in rat synaptosomes [37]. Compound **120** demonstrated remarkable antidepressant activity in vitro, by inhibiting norepinephrine reuptake in rat brain synaptosomes [38]. Simultaneously, seventeen new 2-(2-phenylethyl)chromones, including compounds **22**, **27**–**29**, **31**–**33**, **68**, **69**, **78**–**80**, **82**–**86**, and eleven new terpenoids, such as **103**–**105** and **110**–**117**, had acetylcholinesterase inhibitive effect [8,20,21,26,28,32,33,34,35]. Above all, neural activity of agarwood is one of the most studied aspects with many active compounds and a promising drug candidate found, which will sustain it as a research hotspot in the future.

#### 2.2.2. Gastrointestinal Regulation

Pharmacological studies showed that agarwood and the leaves of *A. sinensis* trees have a gastrointestinal regulating effect. Our studies demonstrated that the agarwood ethanol extract significantly improved intestinal peristalsis, enhanced gastric emptying, and inhibited gastric ulcer [60]. Li et al. reported that the ethanol extract of agarwood and *A. sinensis* leaves enhanced intestinal propulsion [61]. Kakina et al. reported that leaves of *A. sinensis* trees induced laxation via acetylcholine receptors on loperamide-induced constipation in mice [62]. The acetone extract of *A. sinensis* leaves had a laxative effect without causing diarrhea, in which genkwanin 5-*O-β*-primeveroside was the active constituent, whereas the methanol extract did not have the laxative effect [63]. The ethanol extract of *A. sinensis* leaves had a laxative effect without causing diarrhea in a rat model of low-fiber diet-induced constipation [64]. Mangiferin and genkwanin 5-*O*-primeveroside were the two major bioactive compounds [65]. Additionally, benzylacetone, an active compound from essential oil, had the effect of enhancing appetite [66,67]. Even though agarwood on alleviating abdominal discomfort has been widely used for centuries, the gastrointestinal regulating effect, especially on a specific disease, is not completely clear.

#### 2.2.3. Antibacterial and Antifungal

The original use of agarwood was for anticorrosive deodorization in ancient China, as well as Southeast Asian countries. In Thailand, agarwood has been used for a long time as a traditional treatment for infectious diseases such as diarrhea and skin diseases [68]. Chen et al. [69] found that agarwood essential oil derived from *A. sinensis*, regardless of whether it originated from artificial or natural agarwood, had inhibitive activities towards *Bacillus subtilis* and *Staphylococcus aureus* [69]. Extracts of agarwood (*A. crassna*), isolated by water distillation, supercritical fluid carbon dioxide, and supercritical fluid carbon dioxide with ethanol as the co-solvent, showed antimicrobial activities against *S. aureus* and *Candida albicans*, but were not against *Escherichia coli* [70]. Sirilak et al. [68] found that an aqueous extract of *A. crassna* leaves possessed an in vitro antibacterial action against *Staphylococcus epidermidis*, causing bacterial cells to swell and distort, inhibiting the biofilm formation, and leading to cell wall rupture. An ethyl acetate soluble fraction of ethanol extract from *A. crassna* exhibited stronger antifungal (*Fusarium solani*) activity than ethanol extract [10]. Additionally, many other compounds had an antibacterial activity, such as compound **27**, exhibiting inhibitory effect against *S. aureus* [8], compound **105** and **107** against both *S. aureus* and *R. solanacearum*, and compound **109** against *S. aureus* [33]. Even though the antibacterial/antifungal effect of agarwood is definite, the inhibited microbial species are not completely known. Therefore, antibacterial spectrum investigation of agarwood should be carried out.

#### 2.2.4. Anti-Inflammatory

Agarwood essential oil has an anti-inflammatory function, significantly reducing the skin thickness, ear weight, oxidative stress, and pro-inflammatory cytokines production in the 12-*O*-tetradecanoylphorobol-13 acetate (TPA)-induced mouse ear inflammation model [71]. The ethanol extract of agarwood also inhibited ear edema induced by xylene, and peritoneal inflammation induced by low concentrative acetic acid in mice [72]. Linalool and the corresponding acetate derivate play a major role in anti-inflammatory activity [73]. An in silico molecular docking study suggests that 10-epi-*γ*-eudesmol, jinkoh-eremol, and agarospirol were preferentially more active than other identified compounds, with strong binding affinity to major anti-inflammatory receptors [71]. Furthermore, many other activity screening results have shown that compounds from agarwood exhibited a potent inhibitory activity against inflammation. Compounds **34**–**42**, **43**, **48**–**51**, **52**–**56**, **58**, **61**–**63**, **95**, **99**, and **145** showed significant inhibition of NO production [22,23,24,25,30,31,43]. Compound **150** showed inhibition activity against polymorphonuclear neutrophil respiratory burst stimulated by phorbol 12-myristate 13-acetate [45]. Compounds **81** and **144** exhibited inhibition of superoxide anion generation [27], and inversely, compounds **139**–**142** exerted enhancing activity on superoxide anion generation [42]. At the same time, compounds **81**, **139**, and **144** showed potent inhibitory activity on elastase release [27,42]. As we all know, inflammation has a close relationship with other diseases, such as immunopathy, metabolic disorders, and neoplasms, so the anti-inflammatory effect of agarwood, in a certain degree, portends the extensive pharmacological activities of agarwood.

#### 2.2.5. Analgesic Effect

Wang et al. [74] found that chloroform extracts of agarwood prolonged the pain threshold induced by hot plate, and reduced the times of writhing reactions. Jinkoh-eremol and agarospirol may be the active compounds, and jinkoh-eremol’s analgesic effect could be blocked by naloxone (a opioid antagonist), whereas agarosporol was weakly effected by naloxone [51]. At the same time, jinkoh-eremol and agarospirol could inhibit D_2_ receptor binding and 5-HT_2A_ receptor binding [51]. Additionally, compound **138** showed strong inhibitory activity in A23178- and antigen-induced degranulation assay, with IC_50_ values of 1.7 nM and 11 nM, respectively [41].

#### 2.2.6. Antiasthma

The antiasthma effect of agarwood has been traditionally used in China, and can be found in the latest Chinese Pharmacopoeia [13]. However, to our knowledge, only one study found that an ethanol extract of agarwood and *A. sinensis* leaves could inhibit asthma induced by histamine phosphate in guinea pig [75].

#### 2.2.7. Cytotoxicity

Agarwood essential oil possesses anticancer activity towards MCF-7 breast cancer cells [76] and HCT 116 colorectal carcinoma cells [77,78,79]. *β*-Caryophyllene, isolated from the essential oil of *A. crassna*, exhibited selective anti-proliferative effects against colorectal cancer cells (IC_50_ 19 μM) and induced apoptosis via nuclear condensation and fragmentation pathways. Additionally, *β*-caryophyllene also showed potent inhibition of clonogenicity, migration, invasion, and spheroid formation in colon cancer cells [80]. Additionally, other activity screening results showed that compounds from agarwood exhibited cytotoxic activity [81], whereas compound **88** suppressed tumor promotion at noncytotoxic concentrations [29].

#### 2.2.8. Anti-Diabetes

Mei et al. [82] found that the ethanol extracts of both agarwood and *A. sinensis* leaves alleviated diabetes induced by mesoxyalyurea in mice. The methanol extract of *A. sinensis* leaves possessed the fast blood glucose activity in rat and glucose uptake transportation by rat adipocytes [83]. Iriflophenone 3-C-*β*-glucoside decreased the fasting blood glucose levels in streptozocin-induced diabetic mice, and enhanced glucose uptake into adipocytes [84]. Compounds **146**–**149** isolated from agarwood had an inhibitive effect on *α*-glucosidase [44].

#### 2.2.9. Antioxidation

The essential oil of agarwood had a protective effect against oxidative damage induced by hydrogen peroxide (H_2_O_2_) in PC12 cells [85]. The aqueous extract of *A. crassna* leaves had radical scavenging capacities determined by 2,2′-azino-bis(3-ethylbenzthiazoline-6-sulphonic acid (ABTS), ferric reducing antioxidant power (FRAP), and 2,2-diphenyl-1-picrylhydrazyl hydrate (DPPH) scavenging assays [68]. A methanol extract of *A. crassna* leaves was also found to have anti-oxidative activities [86]. The 100% (*v/v*) ethanol extract exhibited the highest DPPH radical scavenging activity among the 0% to 100% (*v/v*) ethanol extracts isolated from *A. crassna* young leaves [87]. *β*-Caryophyllene displayed strong antioxidant effects determined by the DPPH and FRAP scavenging methods [80]. Other compounds **28**, **35**, and **144**, isolated from agarwood, also showed an anti-oxidative effect [8,22,27].

#### 2.2.10. Others

A methanol extract of *A. crassna* leaves significantly reduced fever (rectal temperature) induced by baker’s yeast at five and six hours after subcutaneous injection in rat [86]. The aqueous extract of *A. malaccensis* leaves was effective on *Trypanosoma evansi* with an IC_50_ value 36.29 ± 1.32 μg/mL, whereas the ethanol extract was relatively weak (IC_50_ = 128.63 ± 6.70 μg/mL) [88]. An ethyl acetate extract of *A. crassna* showed an anti-ischemic effect by attenuation of P38-MAPK activation [89].

## 3. Conclusions

Among the 154 new compounds identified from *Aquilaria* plants, 2-(2-phenylethyl)-4*H*-chromen-4-one derivatives and sesquiterpenes account for 57% and 35%, respectively, where most of the new compounds, accounting for 89%, were isolated from *A. sinensis*. Generally, agarwood originating from different *Aquilaria* plants share some common compounds, but still have several different compounds [14]. In addition, there are at least 19 species of *Aquilaria* plants producing agarwood, which means that large quantities of new compounds need to be explored in agarwood and *Aquilaria* plants. The chemical components of agarwood are diverse and complex, contributing to the diversity of bioactivity and pharmacology, including neural activity, gastrointestinal regulation, antibacterial, anti-inflammation, and cytotoxicity. Based on the specific disease and target, illuminating the active ingredients and compounds of agarwood should be carried out, which may not only contribute to the understanding of the scientific nature of the traditional agarwood application, but also benefit the new drug research and agarwood product development.

## Figures and Tables

**Table:** 

NO.	R^1^	R^2^	R^3^	R^4^	R^5^	R^6^	R^7^	R^8^
**1**	H	OH	OCH_3_	H	H	H	OH	OCH_3_
**2**	H	OCH_3_	OCH_3_	H	H	H	OH	OCH_3_
**3**	H	OCH_3_	OH	H	H	H	OH	OCH_3_
**4**	H	OCH_3_	OCH_3_	H	H	H	OCH_3_	OH
**5**	H	OH	OH	H	H	H	H	OCH_3_
**6**	H	OH	OCH_3_	H	H	H	H	OH
**7**	H	OH	H	OH	H	H	OH	OCH_3_
**9**	OH	OH	OH	OH	H	H	OH	OCH_3_
**10**	H	OH	H	Cl	H	H	H	H
**11**	H	OH	H	Cl	H	H	H	OCH_3_
**12**	H	OCH_3_	OH	H	H	H	H	OCH_3_
**20**	H	OH	OCH_3_	H	H	H	H	OCH_3_
**21**	H	OH	H	H	H	H	OCH_3_	OCH_3_
**22**	H	OH	H	OH	H	H	H	OCH_3_
**23**	H	OH	H	Cl	H	H	OH	OCH_3_
**24**	OCH_3_	OH	H	H	H	H	OH	OCH_3_
**25**	H	OCH_3_	OCH_3_	H	*α*-OH	H	H	H
**26**	H	OCH_3_	OCH_3_	H	*β*-OH	H	H	H
**27**	H	OCH_3_	H	H	H	H	OH	OCH_3_
**28**	OH	OCH_3_	H	H	H	H	OH	OCH_3_
**40**	H	OH	OCH_3_	H	H	H	OCH_3_	OH
**41**	H	OCH_3_	H	H	H	H	H	OH
**42**	H	OH	H	H	H	H	OH	OCH_3_
**43**	OH	H	OCH_3_	H	H	H	H	OCH_3_
**44**	OH	OCH_3_	H	OH	H	H	H	H
**46**	H	OCH_3_	H	H	H	OH	OH	OH
**47**	OH	OCH_3_	OCH_3_	H	H	H	H	OCH_3_
**81**	H	OCH_3_	OH	H	H	H	H	H
**82**	H	H	H	H	H	H	OH	OCH_3_
**83**	H	H	H	H	H	H	OCH_3_	OH
**84**	H	H	H	H	H	OH	H	OCH_3_
**85**	H	H	H	H	H	H	H	OH
**86**	H	H	H	H	H	H	OH	H
**87**	H	H	H	H	H	OH	H	H
**88**	H	H	H	H	OH	H	H	H

**Table:** 

NO.	R^1^	R^2^	R^3^	R^4^	R^5^	R^6^
**16**	*α*-OH	*β*-OH	*β*-OH	*α*-Cl	H	OCH_3_
**17**	*α*-OH	*β*-OH	*β*-OH	*α*-Cl	OH	OCH_3_
**18**	*α*-OH	*α*-OH	*β*-OH	H	H	H
**19**	*α*-OH	*α*-OH	*β*-OH	H	H	OCH_3_
**34**	*α*-OH	*α*-OH	*α*-OH	*β*-OH	OH	OCH_3_
**35**	*α*-OH	*α*-OH	*α*-OH	*β*-OH	H	H
**36**	*α*-OH	*α*-OH	*α*-OH	*β*-OH	H	OCH_3_
**37**	*α*-OH	*β*-OH	*α*-OH	*β*-OH	OH	OCH_3_
**38**	*α*-OH	*β*-OH	*β*-OH	*α*-OH	OH	OCH_3_
**39**	*α*-OH	*β*-OH	*β*-OH	*α*-OH	H	OH
**48**	*β*-OH	*β*-OH	*β*-OH	*α*-Cl	H	OCH_3_
**49**	*α*-OH	*α*-OH	*α*-OH	*α*-Cl	H	H
**50**	*β*-OH	*β*-OH	*β*-OH	*β*-Cl	H	OCH_3_
**51**	*β*-OH	*β*-OH	*β*-OH	H	OCH_3_	OH
**68**	*α*-OCH_3_	*β*-OH	*β*-OH	*α*-OH	H	OCH_3_
**69**	*β*-OCH_3_	*α*-OH	*α*-OH	*β*-OH	H	OCH_3_
**70**	*α*-OCH_3_	*β*-OH	*β*-OH	*α*-OH	OH	OCH_3_
**71**	*α*-OCH_3_	*β*-OH	*β*-OH	*α*-Cl	H	OCH_3_
**72**	*α*-OH	*β*-OH	*β*-OH	*α*-Cl	H	OCH_3_
**73**	*α*-OCH_3_	*α*-OH	*α*-OH	*β*-OH	H	OCH_3_
**74**	*β*-OCH_3_	*β*-OH	*β*-OH	*α*-OH	H	OCH_3_
**75**	*α*-OCH_3_	*α*-OH	*α*-OH	*β*-OH	OH	OCH_3_
**76**	*α*-OCH_3_	*α*-OH	*α*-OH	*β*-Cl	H	OCH_3_
**77**	*α*-OCH_3_	*α*-OH	*α*-OH	*β*-Cl	OH	OCH_3_

**Table 1:** Chemical constituents of agarwood originating from the genus *Aquilaria.*

No.	Compound Class and Name	Source or Origin	Extraction *	Ref.
	**2-(2-Phenylethyl)chromones**			
**1**	7-Hydroxy-6-methoxy-2-[2-(3′-hydroxy-4′-ethoxyphenyl)ethyl]chromone	*A. sinensis* (China)	EtOH	[16]
**2**	6,7-Dimethoxy-2-[2-(4′-hydroxy-3′-methoxyphenyl) ethyl]chromone	*A. sinensis* (China)	EtOH	[16]
**3**	6,7-Dihydroxy-2-[2-(4′-methoxyphenyl)ethyl]chromone	*A. sinensis* (China)	EtOH	[16]
**4**	6-Hydroxy-7-methoxy-2-[2-(4′-hydroxyphenyl)ethyl]chromone	*A. sinensis* (China)	EtOH	[16]
**5**	6,8-Dihydroxy-2-[2-(3′-hydroxy4′-methoxyphenyl)ethyl]chromone	*A. sinensis* (China)	EtOH	[16]
**6**	6-Hydroxy-2-[2-(4′-hydroxy-3′-methoxyphenyl)ethenyl]chromone	*A. sinensis* (China)	EtOH	[16]
**7**	6-Hydroxy-7-methoxy2-[2-(3′-hydroxy-4′-methoxyphenyl)ethyl]chromone	*A. sinensis* (China)	EtOH	[16]
**8**	6,7-Dimethoxy-2-[2-(3′-hydroxy-4′-xyphenyl)ethyl]chromone	*A. sinensis* (China)	EtOH	[16]
**9**	5,6,7,8-Tetrahydroxy-2-(3-hydroxy-4-methoxyphenethyl)-5,6,7,8-tetrahydro-4*H*-chromen-4-one	*A. sinensis* (China)	EtOH–H_2_O	[17]
**10**	8-Chloro-6-hydroxy-2-(2-phenylethyl)chromen-4-one	*A. sinensis* (China)	EtOH–EtOAc	[18]
**11**	8-Chloro-6-hydroxy-2-[2-(4-methoxyphenyl)ethyl]chromen-4-one	*A. sinensis* (China)	EtOH–EtOAc	[18]
**12**	Rel-(5*R*,6*S*,7*R*)-5,6,7,8-Tetrahydro-5,6,7-trihydroxy-2-(2-phenylethyl)-4*H*-1-benzopyran-4-one	*A. malaccensis* (Laos)	EtOH–*n*-BuOH	[19]
**13**	Rel-(5*R*,6*S*,7*R*)-5,6,7,8-Tetrahydro-5,6,7-trihydroxy-2-[2-(4-methoxyphenyl)ethyl]-4*H*-1-benzopyran4-one	*A. malaccensis* (Laos)	EtOH–*n*-BuOH	[19]
**14**	7-Hydroxy-6-methoxy-2-[2-(4-methoxyphenyl)ethyl]-4*H*-1-benzopyran-4-one	*A. malaccensis* (Laos)	EtOH–*n*-BuOH	[19]
**15**	Rel-(1a*R*,2*R*,3*R*,7b*S*)-1*α*,2,3,7*β*-Tetrahydro-2,3-dihydroxy-5-[2-(4-methoxyphenyl)ethyl]-7*H*-oxireno[f] [1]benzopyran-7-one	*A. malaccensis* (Laos)	EtOH–*n*-BuOH	[19]
**16**	Rel-(1a*R*,2*R*,3*R*,7b*S*)-1*α*,2,3,7*β*-Tetrahydro-2,3-dihydroxy-5-(2-phenylethyl)-7*H*-oxireno[f] [1]benzopyran-7-one	*A. malaccensis* (Laos)	EtOH–*n*-BuOH	[19]
**17**	Rel-(1a*R*,2*R*,3*R*,7b*S*)-1*α*,2,3,7*β*-Tetrahydro-2,3-dihydroxy-5-[2-(3-hydroxy-4-methoxyphenyl)ethyl]-7*H*-oxireno[f] [1]benzopyran-7-one	*A. malaccensis* (Laos)	EtOH–*n*-BuOH	[19]
**18**	Rel-(5*R*,6*S*,7*S*,8*R*)-8-Chloro-5,6,7,8-tetrahydro-5,6,7-trihydroxy-2-[2-(4-methoxyphenyl)ethyl]-4*H*-1-benzopyran-4-one	*A. malaccensis* (Laos)	EtOH–*n*-BuOH	[19]
**19**	Rel-(5*R*,6*S*,7*S*,8*R*)-8-Chloro-5,6,7,8-tetrahydro-5,6,7-trihydroxy-2-[2-(3-hydroxy-4-methoxyphenyl)ethyl]-4*H*-1-benzopyran-4-one	*A. malaccensis* (Laos)	EtOH–*n*-BuOH	[19]
**20**	6-Hydroxy-7-methoxy-2-[2-(4-methoxyphenyl)ethyl]chromone	*A. sinensis* (China)	EtOH–EtOAc	[20]
**21**	6-Hydroxy-2-[2-(3,4-dimethoxyphenyl)ethyl]chromone	*A. sinensis* (China)	EtOH–EtOAc	[20]
**22**	6,8-Dihydroxy-2-[2-(4-methoxyphenyl)ethyl]chromone	*A. sinensis* (China)	EtOH–EtOAc	[20]
**23**	8-Chloro-6-hydroxy-2-[2-(3-methoxy-4-hydroxyphenyl)ethyl]chromone	*A. sinensis* (China)	EtOH–EtOAc	[20]
**24**	5-Methoxy-6-hydroxy-2-[2-(3-methoxy-4-hydroxyphenyl)ethyl]chromone	*A. sinensis* (China)	EtOH–EtOAc	[20]
**25**	(*R*)-6,7-Dimethoxy-2-(2-hydroxy-2-phenylethyl)chromone	*A. sinensis* (China)	EtOH–EtOAc	[20]
**26**	(*S*)-6,7-Dimethoxy-2-(2-hydroxy-2-phenylethyl)chromone	*A. sinensis* (China)	EtOH–EtOAc	[20]
**27**	6-Methoxy-2-[2-(3-hydroxy-4-methoxyphenyl)ethyl]chromone	*A. sinensis* (China)	EtOH–EtOAc	[8]
**28**	5-Hydroxy-6-methoxy-2-[2-(3-hydroxy-4-methoxyphenyl)ethyl]chromone	*A. sinensis* (China)	EtOH–EtOAc	[8]
**29**	5,6-Epoxy-7*β*-hydroxy-8*β*-methoxy-2-(2-phenylethyl)chromone	*A. sinensis* (China)	EtOH–EtOAc	[8]
**30**	(5*S*,6*R*,7*S*,8*R*)-2-[2-(4-Methoxyphenyl)ethyl]-5,6,7-trihydroxy-5,6,7,8-tetrahydro-8-{6-methoxy-2-[2-(3‴-methoxy-4‴-hydroxypheny)ethyl]chromonyl-7-oxy}chromone	*A. sinensis* (China)	EtOH–EtOAc	[21]
**31**	(5*S*,6*R*,7*S*,8*R*)-2-[2-(4-Methoxyphenyl)ethyl]-5,6,7-trihydroxy-5,6,7,8-Tetrahydro-8-{2-[2-(4‴-methoxyphenyl)ethyl]chromonyl-6-oxy}chromone	*A. sinensis* (China)	EtOH–EtOAc	[21]
**32**	(5*S*,6*R*,7*S*,8*R*)-2-(2-Phenylethyl)-5,6,7-trihydroxy-5,6,7,8-tetrahydro-8-[2-(2-phenylethyl)chromonyl-6-oxy]chromone	*A. sinensis* (China)	EtOH–EtOAc	[21]
**33**	(5*R*,6*R*,7*R*,8*S*)-2-(2-Phenylethyl)-5,6,7-trihydroxy-5,6,7,8-tetrahydro-8-[2-(2-phenylethyl)chromonyl-6-oxy]chromone	*A. sinensis* (China)	EtOH–EtOAc	[21]
**34**	Aquilarone A	*A. sinensis* (China)	EtOH–CHCl_3_	[22]
**35**	Aquilarone B	*A. sinensis* (China)	EtOH–CHCl_3_	[22]
**36**	Aquilarone C	*A. sinensis* (China)	EtOH–CHCl_3_	[22]
**37**	Aquilarone D	*A. sinensis* (China)	EtOH–CHCl_3_	[22]
**38**	Aquilarone E	*A. sinensis* (China)	EtOH–CHCl_3_	[22]
**39**	Aquilarone F	*A. sinensis* (China)	EtOH–CHCl_3_	[22]
**40**	Aquilarone G	*A. sinensis* (China)	EtOH–CHCl_3_	[22]
**41**	Aquilarone H	*A. sinensis* (China)	EtOH–CHCl_3_	[22]
**42**	Aquilarone I	*A. sinensis* (China)	EtOH–CHCl_3_	[22]
**43**	5-Hydroxy-7-methoxy-2-[2-(4-methoxyphenyl)ethyl]chromone	*A. sinensis* (China)	EtOH–CH_2_Cl_2_	[23]
**44**	5,8-Dihydroxy-6-methoxy-2-(2-phenylethyl)chromone	*A. sinensis* (China)	EtOH–CH_2_Cl_2_	[23]
**45**	5*α*,6*α*-Epoxy-7*β*,8*α*,30-trihydroxy-40-methoxy-2-(2-phenylethyl)chromone	*A. sinensis* (China)	EtOH–CH_2_Cl_2_	[23]
**46**	6-Methoxy-2-[2-(20,30,40-trihydroxy)phenyl)ethyl]chromone	*A. sinensis* (China)	EtOH–CH_2_Cl_2_	[23]
**47**	5-Hydroxy-6,7-dimethoxy-2-[2-(4′-methoxyphenyl)ethyl]chromone	*A. sinensis* (China)	EtOH–EtOAc	[24]
**48**	(5*R*,6*R*,7*R*,8*S*)-8-Chloro-5,6,7-trihydroxy-2-(4-methoxyphenethyl)-5,6,7,8-tetrahydrochromone	*A. sinensis* (China)	EtOH–EtOAc	[24]
**49**	(5*S*,6*S*,7*S*,8*S*)-8-Chloro-5,6,7-trihydroxy-2-(2-phenylethyl)-5,6,7,8-tetrahydrochromone	*A. sinensis* (China)	EtOH–EtOAc	[24]
**50**	(5*R*,6*R*,7*R*,8*R*)-8-Chloro-5,6,7-trihydroxy-2-(4-methoxyphenethyl)-5,6,7,8-tetrahydrochromone	*A. sinensis* (China)	EtOH–EtOAc	[24]
**51**	(5*R*,6*S*,7*S*)-5,6,7-Trihydroxy-2-(4-hydroxy-3-methoxyphenethyl)-5,6,7,8-tetrahydrochromone	*A. sinensis* (China)	EtOH–EtOAc	[24]
**52**	(5*S*,6*R*,7*S*,8*R*) Aquisinenone A	*A. sinensis* (China)	EtOH–EtOAc	[25]
**53**	(5*R*,6*S*,7*R*,8*S*) Aquisinenone A	*A. sinensis* (China)	EtOH–EtOAc	[25]
**54**	(−)-4′-Methoxyaquisinenone A	*A. sinensis* (China)	EtOH–EtOAc	[25]
**55**	(5*R*,6*S*,7*R*,8*S*) Aquisinenone B	*A. sinensis* (China)	EtOH–EtOAc	[25]
**56**	(5*S*,6*R*,7*S*,8*R*)Aquisinenone B	*A. sinensis* (China)	EtOH–EtOAc	[25]
**57**	(−)-6″-Hydroxyaquisinenone B	*A. sinensis* (China)	EtOH–EtOAc	[25]
**58**	(+)-6″-Hydroxy-4′,4‴-dimethoxyaquisinenone B	*A. sinensis* (China)	EtOH–EtOAc	[25]
**59**	(5*R*,6*S*,7*R*,8*S*)Aquisinenone C	*A. sinensis* (China)	EtOH–EtOAc	[25]
**60**	(5*S*,6*R*,7*S*,8*R*)Aquisinenone C	*A. sinensis* (China)	EtOH–EtOAc	[25]
**61**	(−)-Aquisinenone D	*A. sinensis* (China)	EtOH–EtOAc	[25]
**62**	(5*R*,6*S*,7*R*,8*S*)4′-Demethoxyaquisinenone D	*A. sinensis* (China)	EtOH–EtOAc	[25]
**63**	(5*S*,6*R*,7*S*,8*R*)4′-Demethoxyaquisinenone D	*A. sinensis* (China)	EtOH–EtOAc	[25]
**64**	(+)-Aquisinenone E	*A. sinensis* (China)	EtOH–EtOAc	[25]
**65**	(−)-Aquisinenone F	*A. sinensis* (China)	EtOH–EtOAc	[25]
**66**	(−)-Aquisinenone G	*A. sinensis* (China)	EtOH–EtOAc	[25]
**67**	(+)-4′-Methoxyaquisinenone G	*A. sinensis* (China)	EtOH–EtOAc	[25]
**68**	Tetrahydrochromone A	*A. sinensis* (China)	EtOH–EtOAc	[26]
**69**	Tetrahydrochromone B	*A. sinensis* (China)	EtOH–EtOAc	[26]
**70**	Tetrahydrochromone C	*A. sinensis* (China)	EtOH–EtOAc	[26]
**71**	Tetrahydrochromone D	*A. sinensis* (China)	EtOH–EtOAc	[26]
**72**	Tetrahydrochromone E	*A. sinensis* (China)	EtOH–EtOAc	[26]
**73**	Tetrahydrochromone F	*A. sinensis* (China)	EtOH–EtOAc	[26]
**74**	Tetrahydrochromone G	*A. sinensis* (China)	EtOH–EtOAc	[26]
**75**	Tetrahydrochromone H	*A. sinensis* (China)	EtOH–EtOAc	[26]
**76**	Tetrahydrochromone I	*A. sinensis* (China)	EtOH–EtOAc	[26]
**77**	Tetrahydrochromone J	*A. sinensis* (China)	EtOH–EtOAc	[26]
**78**	Tetrahydrochromone K	*A. sinensis* (China)	EtOH–EtOAc	[26]
**79**	Tetrahydrochromone L	*A. sinensis* (China)	EtOH–EtOAc	[26]
**80**	Tetrahydrochromone M	*A. sinensis* (China)	EtOH–EtOAc	[26]
**81**	7-Hydroxyl-6-methoxy-2-(2-phenylethyl)chromone	*A. sinensis* (China)	EtOH–EtOAc	[27]
**82**	Qinanone A	*A. sinensis* (China)	EtOH–Et_2_O	[28]
**83**	Qinanone B	*A. sinensis* (China)	EtOH–Et_2_O	[28]
**84**	Qinanone C	*A. sinensis* (China)	EtOH–Et_2_O	[28]
**85**	Qinanone D	*A. sinensis* (China)	EtOH–Et_2_O	[28]
**86**	Qinanone E	*A. sinensis* (China)	EtOH–Et_2_O	[28]
**87**	Qinanone G	*A.sinensis* (China)	EtOH–Et_2_O	[28]
**88**	2-(2-Hydroxy-2-phenylethyl)-4*H*-chromen-4-one	*A. filaria* (Japan)	EtOH–MeOH	[29]
	**Terpenoids**			
**89**	(+)-9*β*-Hydroxyeudesma-4,11(13)-dien-12-al	*A.sinensis* (China)	EtOH–petroleum ether	[30]
**90**	(+)-Eudesma-4,11(13)-dien-8*α*,9*β*-diol	*A.sinensis* (China)	EtOH–petroleum ether	[30]
**91**	(+)-8*α*-Hydroxyeudesma-3,11(13)-dien-14-al	*A.sinensis* (China)	EtOH–petroleum ether	[30]
**92**	(+)-Eudesma-3,11(13)-dien-8*α*,9*β*-diol	*A.sinensis* (China)	EtOH–petroleum ether	[30]
**93**	(+)-Eudesma-4(14),11(13)-dien-8*α*,9*β*-diol	*A.sinensis* (China)	EtOH–petroleum ether	[30]
**94**	(4*R*,5*R*,7*S*,9*S*,10*S*)-(−)-Eudesma-11(13)-en-4,9-diol	*A.sinensis* (China)	EtOH–petroleum ether	[30]
**95**	(+)-9*β*,10*β*-Epoxyeremophila-11(13)-en	*A.sinensis* (China)	EtOH–petroleum ether	[30]
**96**	(+)-11-Hydroxyvalenc-1(10),8-dien-2-one	*A.sinensis* (China)	EtOH–petroleum ether	[30]
**97**	(−)-Eremophila-9-en-8*β*,11-diol	*A.sinensis* (China)	EtOH–petroleum ether	[30]
**98**	1,10-Dioxo-4*H*-5*H*-7*H*-11*H*-1,10-secoguaia-2(3)-en-12,8-olide	*A. sinensis* (China)	EtOH	[31]
**99**	1-Hydroxy-4*H*-5*H*-7*H*-11*H*-8,9-secoguaia-9(10)-en-8,12-olide	*A. sinensis* (China)	EtOH	[31]
**100**	1-Hydroxy-4*α*,10*α*-dimethyl-5*H*-octahydro-azulen-8-one	*A. sinensis* (China)	EtOH	[31]
**101**	1*α*-Hydroxy-4*α*,10*α*-dimethyl-5*βH*-octahydro-azulen-8-one	*A. sinensis* (China)	EtOH	[31]
**102**	4-Hydroxyl-baimuxinol	*A. sinensis* (China)	EtOH–Et_2_O	[32]
**103**	7*β*-*H*-9(10)-ene-11,12-Epoxy-8-oxoeremophilane	*A. sinensis* (China)	EtOH–Et_2_O	[32]
**104**	7*α*-*H*-9(10)-ene-11,12-Epoxy-8-oxoeremophilane	*A. sinensis* (China)	EtOH–Et_2_O	[32]
**105**	(5*S*,7*S*,9*S*,10*S*)-(+)-9-Hydroxy-selina-3,11-dien-12-al	*A. sinensis* (China)	EtOH–EtOAc	[33]
**106**	(5*S*,7*S*,9*S*,10*S*)-(−)-9-Hydroxy-selina-3,11-dien-14-al	*A. sinensis* (China)	EtOH–EtOAc	[33]
**107**	(5*S*,7*S*,9*S*,10*S*)-(+)-9-Hydroxy-eudesma-3,11(13)-dien-12-methyl ester	*A. sinensis* (China)	EtOH–EtOAc	[33]
**108**	(7*S*,9*S*,10*S*)-(+)-9-Hydroxy-selina-4,11-dien-14-al	*A. sinensis* (China)	EtOH–EtOAc	[33]
**109**	(7*S*,8*S*,10*S*)-(+)-8,12-Dihydroxy-selina-4,11-dien-14-al	*A. sinensis* (China)	EtOH–EtOAc	[33]
**110**	Qinanol A	*A. sinensis* (China)	EtOH–Et_2_O	[34]
**111**	Qinanol B	*A. sinensis* (China)	EtOH–Et_2_O	[34]
**112**	Qinanol C	*A. sinensis* (China)	EtOH–Et_2_O	[34]
**113**	Qinanol D	*A. sinensis* (China)	EtOH–Et_2_O	[34]
**114**	Qinanol E	*A. sinensis* (China)	EtOH–Et_2_O	[34]
**115**	Qinanol F	*A. sinensis* (China)	EtOH–Et_2_O	[34]
**116**	3-oxo-7-Hydroxylholosericin A	*A. sinensis* (China)	EtOH–EtOAc	[35]
**117**	1,5,8,12-Diepoxy-guaia-12-one	*A. sinensis* (China)	EtOH–EtOAc	[35]
**118**	(+)-8*β*-Hydroxy-longicamphenylone	*A. sinensis* (China)	EtOH–petroleum ether	[37]
**119**	11*β*-Hydroxy-13-isopropyl-dihydrodehydrocostus lactone	*A. sinensis* (China)	EtOH–petroleum ether	[37]
**120**	Aquilarabietic acid A	*A. sinensis* (China)	EtOH	[38]
**121**	Aquilarabietic acid B	*A. sinensis* (China)	EtOH	[38]
**122**	Aquilarabietic acid C	*A. sinensis* (China)	EtOH	[38]
**123**	Aquilarabietic acid D	*A. sinensis* (China)	EtOH	[38]
**124**	Aquilarabietic acid E	*A. sinensis* (China)	EtOH	[38]
**125**	Aquilarabietic acid F	*A. sinensis* (China)	EtOH	[38]
**126**	Aquilarabietic acid G	*A. sinensis* (China)	EtOH	[38]
**127**	Aquilarabietic acid H	*A. sinensis* (China)	EtOH	[38]
**128**	Aquilarabietic acid I	*A. sinensis* (China)	EtOH	[38]
**129**	Aquilarabietic acid J	*A. sinensis* (China)	EtOH	[38]
**130**	Aquilarabietic acid K	*A. sinensis* (China)	EtOH	[38]
**131**	Aquilarin B	*A. sinensis* (China)	EtOH–EtOAc	[39]
**132**	Aquilanol A	*A. malaccensis* (Laos)	EtOH–Et_2_O	[36]
**133**	Aquilanol B	*A. malaccensis* (Laos)	EtOH–Et_2_O	[36]
**134**	Daphnauranol D	*A. malaccensis* (Laos)	EtOH–Et_2_O	[36]
**135**	Chamaejasmone E	*A. malaccensis* (Laos)	EtOH–Et_2_O	[36]
**136**	Aquilacallane A	*A. sinensis* (China)	EtOH–EtOAc	[40]
**137**	Aquilacallane B	*A. sinensis* (China)	EtOH–EtOAc	[40]
**138**	Aquimavitalin	*A. malaccensis* (Taiwan)	EtOH–EtOAc	[41]
**139**	12-O-(2′*E*,4′*E*)-6-oxohexa-2′,4′-Dienoylphorbol-13-acetate	*A. malaccensis* (Taiwan)	EtOH–EtOAc	[42]
**140**	12-Deoxy-13-O-acetylphorbol-20-(9′*Z*)-octadecenoate	*A. malaccensis* (Taiwan)	EtOH–EtOAc	[42]
**141**	12-*O*-(2′*E*,4′*E*)-6′,7′-(erythro)-dihydroxytetradeca-2′,4′-dienoylphorbol-13-acetate.	*A. malaccensis* (Taiwan)	EtOH–EtOAc	[42]
**142**	12-*O*-(2′*E*,4′*E*)-6′,7′-(threo)-dihydroxytetradeca-2′,4′-dienoylphorbol-13-acetate.	*A. malaccensis* (Taiwan)	EtOH–EtOAc	[42]
	**Flavonoids**			
**143**	4′-*O*-Geranyltricin	*A. sinensis* (Taiwan)	EtOH–EtOAc	[27]
**144**	3′-*O*-Geranylpolloin	*A. sinensis* (Taiwan)	EtOH–EtOAc	[27]
**145**	Aquisiflavoside	*A. sinensis* (China)	EtOH–*n*-BuOH	[43]
**146**	Aquilarisinin	*A. sinensis* (China)	EtOH–*n*-BuOH and EtOAc	[44]
**147**	Aquilarisin	*A. sinensis* (China)	EtOH–*n*-BuOH and EtOAc	[44]
**148**	Aquilarixanthone	*A. sinensis* (China)	EtOH–*n*-BuOH and EtOAc	[44]
**149**	Hypolaetin 5-*O*-*β*-*D*-glucuronopyranoside	*A. sinensis* (China)	EtOH–*n*-BuOH and EtOAc	[44]
**150**	7-*β*-*D*-Glucoside of 5-*O*-methylapigenin	*A. sinensis* (China)	EtOH–*n*-BuOH	[45]
	**Others**			
**151**	Aquilarinoside A	*A. sinensis* (China)	EtOH–*n*-BuOH	[45]
**152**	Aquilarin A	*A. sinensis* (China)	EtOH–EtOAc	[46]
**153**	(9*S*) Megastigma-4,7-diene-2,3,9-triol-9-*O-β-D*-glucopyranoside	*A. sinensis* (China)	EtOH–*n*-BuOH	[47]
**154**	(9*S*) Megastigma-4(13),7-diene-3,6,9-triol-9-*O-β-D*-glucopyranoside	*A. sinensis* (China)	EtOH–*n*-BuOH	[47]

* The first one or two solvents used to extract before the separation on columns. Ethanol: EtOH; ethyl acetate: EtOAc; *n*-butyl alcohol: *n*-BuOH; diethyl ether: Et_2_O; chloroform: CHCl_3_; and dichloromethane: CH_2_Cl_2_.
